# Differentiation of Rat Adipose-Derived Stem Cells into Parathyroid-Like Cells

**DOI:** 10.1155/2020/1860842

**Published:** 2020-06-12

**Authors:** Ping Zhang, Hao Zhang, Wenwu Dong, Zhihong Wang, Yuan Qin, Changhao Wu, Qi Dong

**Affiliations:** ^1^Department of General Surgery, The First Hospital of China Medical University, Shenyang, Liaoning Province 110001, China; ^2^Department of General Surgery, The People's Hospital of China Medical University, Shenyang, Liaoning Province 110016, China

## Abstract

**Background:**

The current treatment for postoperative hypoparathyroidism has shortcomings, such as repeated blood monitoring for dosage adjustment, uncertain long-term efficacy, and the high price of recombinant parathyroid hormone therapy. Adipose-derived stem cells can undergo adipogenic and osteogenic differentiation in vitro and are considered a novel source of parathyroid-like cells, but the idea lacks theoretical basis and feasibility. We aimed at establishing a protocol for differentiating adipose-derived stem cells into parathyroid-like cells for treating hypoparathyroidism.

**Materials:**

/

**Methods:**

Adipose-derived stem cells were isolated and purified from the inguinal adipose tissue of Sprague Dawley rats. Adipogenic differentiation and osteogenic differentiation of the cells were identified by oil red O and alizarin red S staining, respectively. The adipose-derived stem cells were stimulated by sonic hedgehog (SHH) and activin A. The differentiation of the adipose-derived stem cells to parathyroid-like cells was confirmed by the detection of parathyroid hormone and the related parathyroid markers.

**Results:**

Adipose-derived stem cells were successfully isolated and purified from the rat adipocytes. The adipogenic and osteogenic differentiation capabilities of the adipose-derived stem cells were determined. SHH and activin A stimulated parathyroid hormone secretion by the adipose-derived stem cells and significantly increased the expression of calcium-sensing receptor (CaSR), parathyroid hormone, and glial cells missing homolog 2 (GCM2) in the cells in a time- and concentration-dependent manner.

**Conclusion:**

We successfully differentiated rat adipose-derived stem cells into parathyroid-like cells, which will pave a new route to curing hypoparathyroidism.

## 1. Introduction

The incidence rate of thyroid cancer has become epidemic worldwide, including China [1, 2]. Complications from surgical removal of the parathyroid are rare, but may occasionally occur following thyroid and parathyroid surgery. The incidences of permanent and transient postthyroidectomy hypocalcemia are 0–3% and 19–38%, respectively [3]. Regardless of whether it is unintentional or therapeutic, loss or damage of parathyroid blood transport results in permanent or transient hypoparathyroidism and may therefore cause hypocalcemia.

Hypoparathyroidism results from low parathyroid hormone (PTH) levels. PTH acts as the major hormone for calcium homeostasis, contains 84 amino acids, and is secreted by the parathyroid glands. The treatment of postoperative hypoparathyroidism is challenging. Although recent research has demonstrated evidence of the efficacy of replacement therapy using synthetic PTH, calcium, and vitamin D preparations, limitations remain, such as repeated blood monitoring to adjust the dosage, the short half-value period, and posttreatment complications [4, 5]. Accordingly, better therapeutic strategies should be reestablished to help postoperative patients with hypoparathyroidism and to minimize potential adverse effects. The transplantation of long-lasting, biocompatible hormone-releasing tissue has been considered the ideal hormone replacement therapy for hypoparathyroidism.

Stem cells are a class of cells with self-renewal and differentiation potential. Recently, attempts have been made to differentiate stem cells into parathyroid-like cells. Bingham et al. first reported the successful differentiation of embryonic mesenchymal stem cells into parathyroid-like cells under the influence of activin A [6]. Woods Ignatoski et al. differentiated parathyroid-like cells from thymocytes and embryonic mesenchymal stem cells in children through activin A and sonic hedgehog (SHH) induction [7, 8]. Green et al. used activin A, Noggin, and SB-431542 to differentiate embryonic mesenchymal stem cells and induced pluripotent stem cells into the endoderm, which could be further differentiated into parathyroid-like cells under the action of SHH or FGF8 (fibroblast growth factor 8) [9]. Park et al. successfully differentiated tonsil-derived mesenchymal stem cells into PTH-releasing cells under the influence of activin A and SHH [10]. In contrast, adipose-derived stem cells (ADSCs) are abundant and easily obtained from human adipose tissue. It has been hypothesized that ADSCs can be differentiated into parathyroid cells, which can treat hypoparathyroidism [11]. Furthermore, we have successfully separated and purified ADSCs. Herein, we intend to explore the protocol for differentiating ADSCs into parathyroid-like cells.

## 2. Materials and Methods

### 2.1. Isolation and Purification of Rat ADSCs

All experiments followed the procedures approved by the Institutional Review Board of China Medical University (no. AF-OG-03-1.1-02). Each experiment was repeated independently at least three times. In total, eighteen specific pathogen-free (SPF) 4-week-old Sprague Dawley rats were anesthetized by intraperitoneal injection of sodium pentobarbital and sacrificed. The abdomen was disinfected with 75% alcohol. Abdominal cavity was opened through low transverse incision. Harvest of rat adipose tissue was performed by lipectomy usually by dissection of the inguinal fat pad. The harvested adipose tissue was washed repeatedly using phosphate-buffered saline (PBS), and the blood vessels and other tissues were removed. The remaining tissue was cut into approximately 1 mm × 1 mm × 1 mm pieces and digested in 0.075% type I collagenase on a rocking bed at 37°C for 30 min. The digestion was stopped with an equal volume of high-glucose Dulbecco's modified Eagle's medium (DMEM; Hyclone, Logan, USA) containing 10% fetal bovine serum (FBS; Gibco-BRL, Gaithersburg, MD). After centrifugation at 309x*g* for 10 min, the precipitate was filtered with 200 mesh screens and then treated with red blood cell lysis buffer for 3 min. The reaction was stopped with high-glucose DMEM containing 10% FBS. After rinsing with PBS, the cells were counted and seeded at a density of 3 × 10^6^ cells per dish (75 cm^2^). The cells were cultured in an incubator with 5% CO_2_ at 37°C for 48 h. At 80% confluence, the cells were digested with 0.25% trypsin for 1 min for passage, with one passage performed every 48 h. Third-passage ADSCs were collected for stem cell determination.

### 2.2. Adipogenic Differentiation and Identification

The cells were cultured in low-glucose DMEM containing 10% FBS, 1 *μ*mol/L dexamethasone (Sigma, St. Louis, MO), 200 *μ*mol/L indomethacin (Sigma, St. Louis, MO), 0.5 mmol/L 3-isobutyl-1-methylxanthine (Sigma, St. Louis, MO), and 10 *μ*mol/L insulin (Sigma, St. Louis, MO). The medium was changed every 2 days, and the duration of adipogenic differentiation was 7, 14, and 21 days for each group. The changes in adipogenic differentiation were observed using oil red O staining under an inverted microscope.

### 2.3. Osteogenic Differentiation and Identification

The cells were cultured in low-glucose DMEM containing 10% FBS, 0.1 *μ*mol/L dexamethasone (Sigma, St. Louis, MO), 50 *μ*mol/L ascorbic acid (Sigma, St. Louis, MO), 10 mmol/L *β*-glycerophosphate (Sigma, St. Louis, MO), and 0.01 *μ*mol/L vitamin D3 (Sigma, St. Louis, MO). The medium was changed every 3 days, and the duration of osteogenic differentiation was 7, 14, and 21 days for each group. The changes in osteogenic differentiation were observed using alizarin red S staining under an inverted microscope.

### 2.4. Protocol for Differentiating ADSCs into Parathyroid-Like Cells

Fifth-passage cells were cultured in Roswell Park Memorial Institute (RPMI)-1640 (Hyclone, Logan, USA) medium containing 5% FBS and 100 ng/mL SHH or 25 ng/mL, 50 ng/mL, or 75 ng/mL activin A. The following indicators were examined at 7, 14, and 21 days: PTH level in the medium and calcium-sensing receptor (CaSR), glial cells missing homolog 2 (GCM2), and PTH in the cells.

### 2.5. Enzyme-Linked Immunosorbent Assay (ELISA)

The culture medium supernatant was collected and the PTH concentration was measured using a PTH detection ELISA kit (Youersheng, Wuhan, China, cat. no. CEA866Ra) according to the manufacturer's protocol. The optical density (OD) value at 450 nm was detected using a microplate reader (BioTek ELx800, BioTek, Winooski, VT, USA).

### 2.6. Real-Time PCR

Real-time PCR was used to quantify the *Casr*, *Gcm2*, and *Pth* mRNA levels. Total RNA was extracted from cells that had been collected and lysed in TRIzol (Invitrogen, Carlsbad, CA, USA) according to the manufacturer's instructions. The extracted RNA, with an OD260/OD280 absorbance ratio between 1.9 and 2.0, was used to prepare complementary DNA (cDNA) using a reverse transcriptase kit (PrimeScript RT Reagent Kit; Takara, Dalian, China). The cDNA synthesis protocol followed the manufacturer's instructions at 37°C for 15 min, followed by 85°C for 5 sec, on an ABI 9700 GeneAmp PCR system (Applied Biosystems, Life Technologies, Grand Island, NY, USA). Transcripts were quantified with a LightCycler 480 real-time PCR system (Roche Diagnostics, Basel, Switzerland) using a SYBR Premix Ex Taq kit (Takara).

### 2.7. Western Blotting

Western blotting was performed to quantify CaSR, GCM2, and PTH protein levels. The cultured cells were washed with ice-cold PBS and lysed in lysis buffer containing protease inhibitor cocktail (KGP 250 kit, Keygen Biotech, Nanjing, China) for 30 min on ice. Protein levels were quantified using the bicinchoninic acid (BCA) method. For each test, 30 *µ*g protein per sample was used for western blotting. The total protein was separated by sodium dodecyl sulfate-polyacrylamide gel electrophoresis. Transfer was conducted at a constant current of 100 mA for 60 min using polyvinylidene difluoride membranes (Millipore, Billerica, MA, USA). Nonspecific binding was blocked using 5% skimmed milk for 2 h at room temperature. Then, the membranes were incubated at 4°C overnight with primary antibodies against CaSR (1 : 500, Bioss, Shanghai, China), GCM2 (1 : 500, Bioss, Shanghai, China), PTH (1 : 500, Boster, Wuhan, China), and *β*-actin (1 : 1000, Wanleibio, Shenyang, China). Subsequently, the membranes were incubated for 2 h with horseradish peroxidase- (HRP-) conjugated goat anti-rabbit secondary antibody (1 : 5000, Wanleibio). The bands were detected with the chemiluminescence method using an ECL Plus Western Blotting Detection System (Thermo Fisher Scientific, Rockford, IL, USA). The relative density of the bands was measured using ImageJ software (Bethesda, MD, USA).

### 2.8. Statistical Analysis

Statistical analyses were performed using SPSS 22.0 (IBM, Chicago, IL, USA). Data are presented as the means ± standard deviation (SD), and statistical analyses were performed using Student's *t*-test or analysis of variance. A difference was considered significant when *p* < 0.05.

## 3. Results

### 3.1. Identification of Adipogenic Differentiation of Rat ADSCs

**Figure 1:**
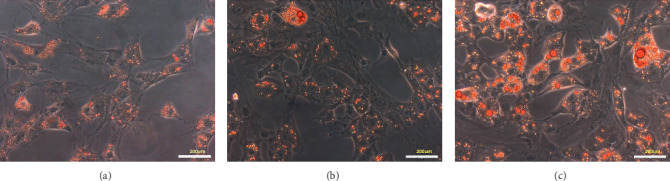
Lipid induced lipid droplet formation in ADSCs. (a) Adding lipid inducer for 7 d, (b) adding lipid inducer for 14 d, and (c) adding lipid inducer for 21 d (scale bar 200* μ*m). ADSCs were stained with oil red O, and the lipid droplets were observed under the inverted microscope.

After incubation with lipid inducer for 7 days (Figure 1(a)), 14 days (Figure 1(b)), and 21 days (Figure 1(c)), the rat ADSCs were stained with oil red O, and the lipid droplets were observed under an inverted microscope. The number of lipid droplets in the cells increased gradually with time, proving that the lipid induction of ADSCs was successful.

### 3.2. Identification of Osteogenic Differentiation of Rat ADSCs

**Figure 2:**
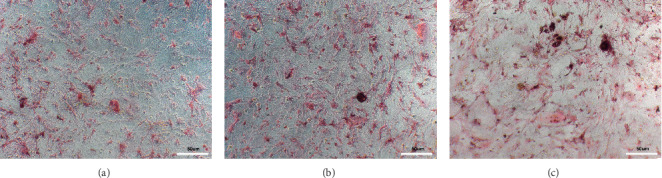
ADSCs induced calcium salt formation after osteogenesis. (a) Adding osteogenic induction fluid for 7 d, (b) adding osteogenic induction fluid for 14 d, and (c) adding osteogenic induction fluid for 21 d (scale bar 50* μ*m). Alizarin red staining and calcium deposition were observed under inverted microscope.

ADSCs were incubated in osteogenic induction medium for 7 days (Figure 2(a)), 14 days (Figure 2(b)), and 21 days (Figure 2(c)). Alizarin red staining and calcium deposition were observed under an inverted microscope. The number of intracellular calcium deposits gradually increased with time, indicating successful induction of osteogenesis in the ADSCs.

### 3.3. Induction of Rat ADSC Differentiation into Parathyroid-Like Cells

**Figure 3:**
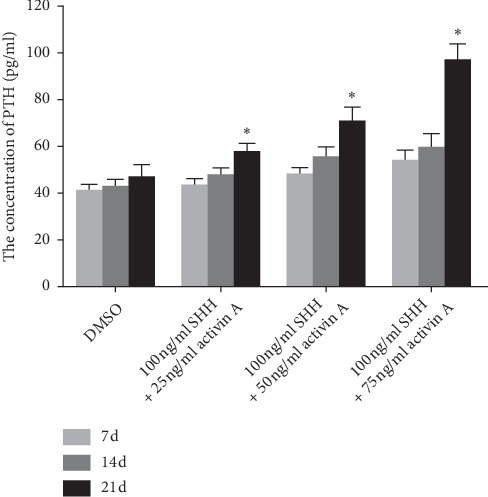
Changes of PTH content after treatment of ADSCs 7 d, 14 d, and 21 d at different concentrations of SHH and activin A.

**Figure 4:**
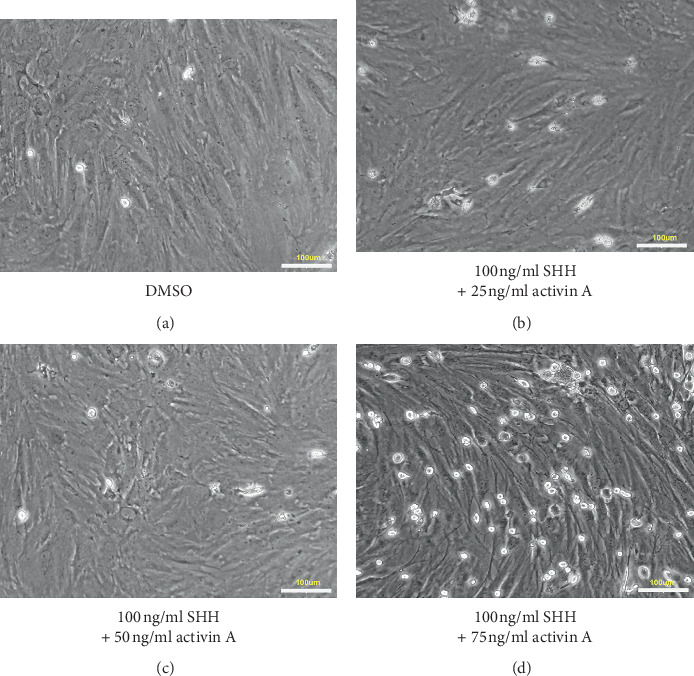
Morphological changes of cells after treatment of ADSCs 21 d with different concentrations of SHH and activin A. (a) Stimulated with DMSO. (b) Stimulated with 100 ng/ml SHH + 25 ng/ml activin A. (c) Stimulated with 100 ng/ml SHH + 50 ng/ml activin A. (d) Stimulated with 100 ng/ml SHH + 75 ng/ml activin A (scale bar 100* μ*m).

The inducible ADSCs were detected in medium containing SHH (100 ng/mL) or activin A (25 ng/mL, 50 ng/mL, and 75 ng/mL) for 7, 14, and 21 days. SHH and activin A had a significant effect on the PTH secretion of the ADSCs, and the secretion was concentration- and time-dependent (Figure 3). The 21-day medium had the highest PTH content, and numerous parathyroid cells were observed under the microscope (Figure 4).

### 3.4. Identification of Parathyroid-Like Cells

**Figure 5:**
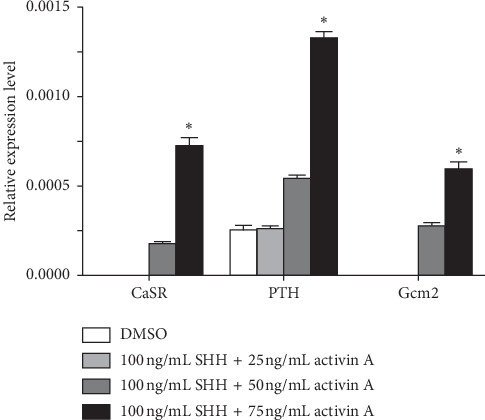
The mRNA expression of CaSR, PTH, and Gcm2 after treatment of ADSCs 21 d with different concentrations of SHH and activin A.

**Figure 6:**
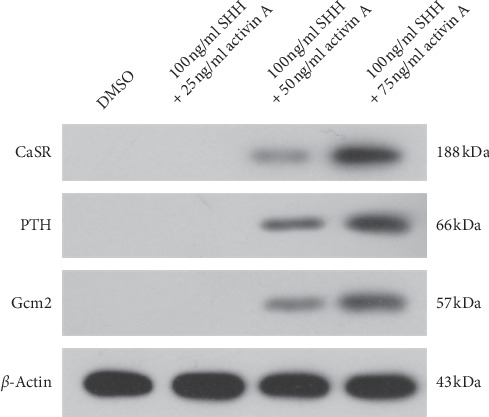
The protein expression of CaSR, PTH, and Gcm2 after treatment of ADSCs 21 d with different concentrations of SHH and activin A.

We used PCR and western blotting to detect the expression of CaSR, PTH, and GCM2 mRNA and protein, respectively, in the 21-day medium. SHH and activin A increased the CaSR, PTH, and GCM2 mRNA and protein levels (Figures 5 and 6). The results show that the ADSCs were differentiated into parathyroid-like cells successfully.

## 4. Discussion

It is believed that human ADSCs can be differentiated into parathyroid-like cells, which can be used as a novel source of parathyroid cells to treat hypoparathyroidism [11]. However, this hypothesis requires evidence. Ours is the first report stating that rat ADSCs in vitro can be differentiated into functional parathyroid-like cells, which provides a reliable theoretical basis for the above hypothesis. Furthermore, we successfully established a detailed protocol for differentiating rat ADSCs into parathyroid-like cells.

Although uncommon, permanent hypoparathyroidism can occasionally occur after thyroidectomy, especially total thyroidectomy. This complication depends on surgical skill, surgeon volume, patients underlying disease process, etc. Autologous transplantation of parathyroid glands has been considered an important means of decreasing the incidence of permanent hypoparathyroidism. Autologous transplantation of at least one parathyroid gland is associated with the low risk of permanent hypoparathyroidism [12, 13]. The parathyroid gland is appropriate for cell transplantation because of its unique anatomical and physiological characteristics. The reasons are as follows: first, each parathyroid cell contains the complete function of the organ and can independently exert the same function as the organ, i.e., they can be transplanted and function without organ construction. Second, transplanting a small amount of parathyroid cells can achieve the normal function of the whole organ. Third, postoperative hypoparathyroidism mostly results from iatrogenic parathyroid damage. There is no immunologic function abnormality, which does not affect the results of the transplantation. However, due to the complexity of the intraoperative situation, surgeons cannot detect injury or miscutting of the parathyroid gland in a timely manner. When hypoparathyroidism occurs after surgery, there are no transplantable seed cells. Therefore, the only possibility for clinical cure of hypoparathyroidism is to find sufficient and safe parathyroid seed cells for autologous transplantation. Accordingly, researchers have attempted to differentiate various stem cells into parathyroid cells for cell transplantation, such as embryonic mesenchymal stem cells and tonsil-derived mesenchymal stem cells [6, 10]. In the present study, we chose ADSCs as a source of stem cells because of the rich source of adipose tissue, easy access to obtain materials with minimal risk, and the relatively mature isolation, expansion, and differentiation techniques. More importantly, ADSCs are multipotent with adipogenic and osteogenic capability and can be successfully differentiated into multiple tissues and cells of the three germ layers [11].

After isolating and purifying the rat ADSCs, we first identified their adipogenic and osteogenic differentiation capability. We found that the intracellular lipid droplets and intracellular calcium deposits increased gradually in a time-dependent manner. Next, we induced the cells. Previous studies have used different induction protocols such as activin A only, activin A + Noggin + SB-431542 + SHH/FGF8, or activin A + SHH [6–10]. Among these protocols, the most frequently used is activin A + SHH. Activin A is a secretory protein of the transforming growth factor-beta (TGF-*β*) superfamily and is widely used in stem cell research. It can mediate the growth and differentiation of multiple stem cells. SHH is also a secreted protein and plays a crucial role in embryonic development. Consistent with the requirement of activin A and SHH for parathyroid development, we also used them as induction reagents and found that activin A and SHH had a significant effect on the PTH secretion and protein expression of the definitive parathyroid cell marker proteins (i.e., CaSR, PTH, and GCM2) by the ADSCs.

PTH is secreted from the parathyroid (chief) cells and acts as the major hormone for regulating calcium homeostasis of the human body. The level of serum ionized calcium is exclusively regulated by PTH. In turn, PTH secretion is regulated by serum calcium levels through CaSR, which is located on the surface of parathyroid cells. Although several genes (e.g., *BMP4*, *NOG*, *SIX1*, and *PBX1*) are involved in parathyroid differentiation, only *GCM2* has been considered to play a functional role in parathyroid development and is a specific parathyroid marker [6]. Therefore, CaSR, PTH, and GCM2 are considered parathyroid markers. Here, we were able to induce rat ADSCs to differentiate into cells that expressed the parathyroid markers CaSR, PTH, and GCM2 and that also secreted PTH.

The present study has several limitations. First, biological function of ADSCs should be further validated and the therapeutic effect of autologous transplantation should be assessed. Second, current research results are limited to animal experiments. There is still a long way to go before clinical application. It is hypothesized that human ADSCs can be differentiated into parathyroid cells for the treatment of hypoparathyroidism [11]. We will make efforts to provide evidence for this hypothesis. In the future study, we will first demonstrate the differentiated ADSCs can adjust PTH upon different Ca^2+^ concentrations in media. Then, we will establish an animal model of hypoparathyroidism and autotransplant the differentiated ADSCs into the rats and measure blood calcium and PTH in tail vein blood of rats to evaluate the therapeutic effect of autologous transplantation. Finally, we will validate this procedure on human adipose tissue.

## 5. Conclusions

Our findings confirm that rat ADSCs can be differentiated into parathyroid-like cells, which lays the foundation for further research in humans. The ultimate goal of our research is to take advantage of the cells from an individual patient with hypoparathyroidism and differentiate them into parathyroid-like cells for replacement therapy.

## Data Availability

Our data are currently unavailable for sharing because further studies are being undertaken.
